# Two Distinctive Phenotypes of AcMNPV Display Different Immune Abilities and Intracellular Destiny

**DOI:** 10.1371/journal.pone.0168939

**Published:** 2016-12-29

**Authors:** Guido N. Molina, Eugenia Tavarone, Oscar Taboga, Paula Molinari

**Affiliations:** 1 Consejo Nacional de Investigaciones Científicas y Técnicas (CONICET), CABA, Argentina; 2 Instituto de Biotecnología, Centro Nacional de Investigaciones Agropecuarias (CNIA), INTA Castelar, Buenos Aires, Argentina; Wuhan Bioengineering Institute, CHINA

## Abstract

The budded phenotype (BV) of the baculovirus AcMNPV has been demonstrated to have strong immunostimulatory properties that are relevant for the development of vaccines and antiviral therapies. Although the occluded phenotype (ODV) shares the main structural proteins and its genome with BV, it has been poorly studied in mammals. In this study, we assessed the capacity of ODV to induce immune responses in mice. In contrast to BVs, ODVs failed to promote the secretion of IFN-gamma, IL-6 and Il-12 and to induce antiviral activity against VSV in the short term. Furthermore, ODVs were unable to induce cellular immunity against a coadministered antigen 7 days after inoculation. By analyzing the interaction of ODVs with BMDCs, we observed that although ODVs entered the cells reaching late and acidic endosomes, they did not induce their maturation. Finally, we also analyzed if BVs and ODVs followed different routes in the cell during the infection. BVs, but not ODVs, colocalized with the protein ovalbumin in compartments with the presence of proteases. The results suggest that structural differences could be responsible for their different destinies in the dendritic cell and this could lead to a different impact on the immune response.

## Introduction

Baculoviruses are dsDNA viruses that infect insects and belong to the *Baculoviridae* family. As the rest of the members of the family, the model species *Autographa californica nucleopolyhedrovirus* is characterized by two distinctive phenotypes with different roles in the larval infection cycle. Firstly, these viruses spread orally as occlusion derived virions (ODVs), which are included into the matrix of occlusion bodies or polyhedra. Polyhedra are dissolved in the alkaline environment of the mid-gut and release ODVs, which in turn infect epithelial cells. Budded virions (BVs), on the other hand, are specialized in infecting internal tissue cells [1]. Both phenotypes have the same major nucleocapsid proteins and the same genome but differ in the origin and composition of their envelopes and in composition of minor structural proteins. Indeed, BVs have an envelope derived from the plasma membrane, whereas ODVs contain a complex envelope derived from the nucleus with nucleocapsids (1 to 15) inside this structure [2].

Baculoviruses have been widely studied and used for insecticide applications and as vectors for protein expression. BVs can transduce a wide variety of mammalian cells with different efficiencies but they are incapable of replicating in these cells [3–5]. In addition, BVs have strong effects on the mammalian immune system. Because of the high frequency of CpG motifs present in its genome, they activate immune cells and stimulate the production of inflammatory cytokines and type I and II interferons (IFNs) via the Toll-like receptor 9 (TLR9)/MyD88-dependent signaling pathway. However, type I and II interferon production is led also via STING cytosolic DNA sensing pathway in some cells [6, 7]. The strong innate immune response elicited by BVs produces an important effect in the strength and the quality of the adaptive immune response.

As mentioned, both phenotypes differ in the composition of surface proteins, although GP64, the major envelope protein of BVs and absent in ODVs, is not *per se* related to the immunostimulatory effects of BVs on murine macrophages [6]. DNA, which contains high frequency of unmethylated CpG motifs, is the major pathogen-associated molecular pattern (PAMP) of baculoviruses [6–9]. Moreover, ODVs can also enter human and non-human cell lines [10, 11]. Today, however, the impact of virions of this phenotype on the mammalian immune response remains elusive.

In this work we studied the immunostimulatory properties of ODVs of Autographa californica nucleopolyhedrovirus (AcMNPV) compared to BVs in a murine model. The findings of this study could help us to better understand and manipulate interactions between baculoviral vectors and immune and non-immune mammalian cells.

## Materials and Methods

### Insect cell cultures and virus amplification and purification

Monolayers of *Spodoptera frugiperda* Sf9 insect cells (Invitrogen, catalogue number B82501) grown in Excell medium culture (Sigma) at 27°C were infected with budded virions of AcMNPV obtained from BaculoGold (Becton Dickinson Argentina S.R.L.). To purify BVs, we infected the cells at a multiplicity of infection (moi) of 0.05. At 5 dpi, the supernatants containing BVs were harvested and cell debris were removed by centrifugation (4,000× g, 15 min, 8°C). Viral stocks were stored at 4°C until use. The moi used for polyhedron purification was 0.5. At 5 dpi, cells were harvested, lysed with SDS 0.5%, and sedimented by centrifugation (5,000× g, 10 min, 4°C). After two washes, one with NaCl 0.5 M and another with distilled water, the stock of polyhedra was stored at -80°C until use. ODVs were released from the polyhedra by dissolving them in 0.1 M sodium carbonate solution. Partially and non-dissolved polyhedra were removed by centrifugation (5,000× g, 10 min, 4°C).

All the material used in the purification procedures was treated with E-TOXATE (Sigma) to eliminate traces of endotoxin.

### Western blot analysis

To analyze the presence of VP39, GP64 and P74 proteins in BVs and ODVs, virions were concentrated and resuspended in Loading buffer (60 mM Tris–HCl pH 6.8, 10% 2-mercaptoethanol, 1% SDS, 0.002% bromophenol blue and 10% glycerol, 1X) and boiled for 10 min. Samples (15 μl) were resolved by 12% SDS polyacrylamide gel electrophoresis. Proteins were blotted onto nitrocellulose membranes and the blots were probed with monoclonal antibodies to VP39 (kindly provided by MA Whitt [12], used 1/2000), GP64 (AcV5, Sigma, used 1/2000) and P74 (N25 8c, kindly provided by P. Faulkner [13], used 1/1000) proteins followed by an alkaline phosphatase-labeled anti-mouse antiserum (Dakkopats, used 1/1000) and an alkaline phosphatase substrate solution (NBT/BCIP, Promega).

### Quantification of viral stocks

To administer an equal number of nucleocapsids of BVs and ODVs, we quantified the two different virions based on the relative amount of VP39. The quantification was performed by comparing the VP39 capsid mass through VP39 intensity in Western blot assays revealed by the monoclonal antibody to VP39. Images were digitalized and analyzed with Image Quant TL 1D software (GE, Healthcare). Additionally, polyhedra were counted in a Neubauer chamber and the content of VP39 was evaluated after dissolving polyhedra with 0.1 M sodium carbonate to release embedded ODVs.

### Electron microscopy

The purified ODVs were adsorbed to formvar-coated copper grids and stained with 2% uranyl acetate. Samples were observed in a Zeiss–EM109T electron microscope.

### Mice

Six to eight week-old female C57BL/6 (H-2^b^) mice from Fundación Facultad de Ciencias Veterinarias (UNLP, La Plata, Argentina) were maintained in our animal facilities under specific pathogen-free conditions. All the experiments were performed in accordance with the Guide to the Care and Use of Experimental Animals by the Canadian Council on Animal Care (Assurance number A5802-01, Office of Laboratory Animal Welfare, NIH). Animal handling and experimental procedures were approved by our Institutional Experimentation Animal Committee (CICUAE-INTA resolution N°68/2014). Isoflurane was used as the anesthetic method. CO_2_ inhalation was used to sacrifice the animals. These methods were selected to minimize animal suffering. No animals became ill or died prior to the experimental endpoint.

### Innate and adaptive immune response: *in vivo* experiments

All C57BL/6 mice were immunized intravenously (i.v.) by single retro-orbital injections to evaluate the innate immune response and the antiviral activity elicited. The animals were injected with BVs, ODVs and polyhedra. All groups were administrated with equal amounts of VP39. A single dose was equivalent to 7x10^7^ TCID50 of BVs. An additional group with four times more of ODVs (ODV 4X) and a control group with PBS were included.

For the *in vivo* killing assay, C57BL/6 mice were injected i.v. with PBS or OVA (1 mg) combined with BVs, ODVs or PBS. BV and ODV doses contained the same amounts of VP39. Splenocytes were obtained from naïve syngeneic mice and half of these cells were pulsed with 10 μg/ml OVA_257–264_ peptide (SIINFEKL), washed and labeled with a high concentration (3 μM) of CFSE (Invitrogen). The non-pulsed half was labeled with a low concentration (0.5 μM) of CFSE as a control. The immunized mice were injected i.v. with a mixture of CFSE^high^- and CFSE^low^-labeled splenocytes at a 1:1 ratio (1×10^7^ cells of each population). After 24 h, the number of CFSE+ cells remaining in the spleen of each mouse was determined using a FACSCalibur flow cytometer and the data were analyzed using the CellQuest (BD Biosciences) software. The percentage of cytotoxic lysis was calculated as [1-(r_control_/r_immune_)]x100, where r is given by the expression of %CFSE^low^/%CFSE^high^ cells for each group of mice.

### Cytokine detection assays

Levels of IL-6, IL-12 p40 and IFN-γ both in culture supernatants and in serum samples were measured by sandwich ELISA according to the manufacturer’s instructions (BD Biosciences).

### Antiviral activity assay

Serial dilutions of serum samples, starting at 1/4, were incubated with IBRS-2 cells (provided by ICT Milstein, Buenos Aires, Argentina) in a 96-well plate (6 replicates per dilution). After 24 h, the supernatants were removed and the cells were infected with vesicular stomatitis virus (VSV) at a moi of 0.01. The infected wells were visualized 24 h later by staining with crystal violet. The antiviral activity was reported as the half maximal inhibitory concentration of the infection (IC_50_), and calculated as the serum concentration (volume/volume percentage, % v/v) that resulted in a 50% reduction of the number of infected wells.

### Statistics

Cytokine measurement and percentage of cytotoxic lysis *in vivo* were performed with one-way ANOVA and Bonferroni posttest as statistical analysis using GraphPad Prism (La Jolla, CA). Values of p<0.05 were considered significant.

### Generation of Bone Marrow-derived Dendritic Cells (BMDCs)

Bone marrow cells were collected from the femurs and tibias of six-week-old female C57BL/6 mice and differentiated to BMDCs by a procedure based on Inaba and colleagues’ protocols [14]. Briefly, 2.5x10^6^ cells were cultured in 100 mm plates containing 10 ml complete RPMI medium (Invitrogen) and 10% of supernatant from the stably transfected GM-CSF-J558 cell line were used to supplement the medium at days 0, 3 and 7. The loosely-adherent BMDCs were used in the experiments at day 8 (>80% of the harvested cells expressed CD11c).

### Analysis of BMDCs maturation markers

For the BMDCs maturation study, 3x10^6^ immature BMDCs were incubated for 18 h with RPMI alone or with BVs, ODVs or polyhedra. All groups were administrated with equal amounts of VP39. The quantity administrated was equivalent to a moi of 10 of BVs. An additional group with ten times more of ODVs (ODV 10X) and another with ten times more of polyhedra (polyhedra 10X) were included.

The BMDCs were labeled using the following monoclonal antibodies: anti-CD11c (HL-3 clone), anti-CD86 (B7.2, PO3.1 clone) and anti-MHC II (I-A/I-E, M5/114.15.2 clone) from eBioscience. The procedure was carried out based on standard protocols [15], using a FACSCalibur flow cytometer. The CellQuest (BD Biosciences) software was used for the analysis. Appropriate isotype controls were included in every experiment.

### Confocal microscopy

Lab-Tek Chambered coverglass dishes (Nunc) or poly-lysine–treated glass slides were used for BMDCs immunofluorescences. The infections were incubated at 37°C for different periods of time. When needed, OVA-FITC (OVA Sigma stained with FITC Sigma), DQ-OVA (Sigma) or LysoTracker Red DND-99 (Life Technologies) was added at the same time as the virus. The cells were then fixed with 2% paraformaldehyde, permeabilized in PBS with 0.05% saponin and 10% bovine serum, and stained with the following antibodies: Mab α-VP39 and anti-LAMP2 (Developmental Studies Hybridoma Bank) followed by staining with Alexa Fluor 488–coupled anti–mouse (Invitrogen) and Cy5-coupled anti-goat (Jackson Immuno Research Inc.), respectively. Slides were analyzed by confocal microscopy Leica-SP5 (from the National University of La Plata).

## Results

### Characterization of ODV and BV phenotypes

**Fig 1:**
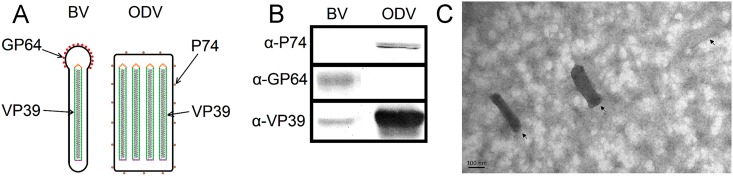
Purified ODVs characterization. (A) Schematic representation of the structure of both baculovirus phenotypes. The main proteins of the capsid and envelope are indicated with arrows. (B) Comparative Western blot analysis between BVs and ODVs. The proteins were detected by specific monoclonal antibodies α-P74, α-GP64 and α-VP39. (C) Transmission electron microscopy of ODVs. Purified ODVs were adsorbed to a copper grid, stained with 2% uranyl acetate and observed at a magnification of x 85,000. The displayed virions are representative of all the ODVs examined. Bar = 100 nm.

The AcMNPV ODVs were purified from late infected cultures of Sf9 cells by dissolving polyhedra and ultracentrifugating the virions derived from occlusion bodies. BVs were obtained from supernatants of infected cells. By Western blots (Wb), we characterized the phenotypical identity of the viral stocks by assessing the presence of the major capsid protein VP39, the major envelope protein GP64 of BV and the major envelope protein P74 of ODVs with specific antibodies. VP39 was present in both phenotypes, whereas P74 and GP64 were exclusively in ODV or BV viral particles, respectively (Fig 1B). Therefore, ODV and BV enveloped viral particles were detectable in the respective viral stocks, without cross-contamination.

Transmission electron microscopy revealed entire and enveloped particles of ODVs, thus demonstrating ODV integrity. The examined virions were in average 78±26 nm in width and 270±21 nm in length and representative virions are shown in Fig 1C.

### ODVs do not stimulate the immune response in mice

**Fig 2:**
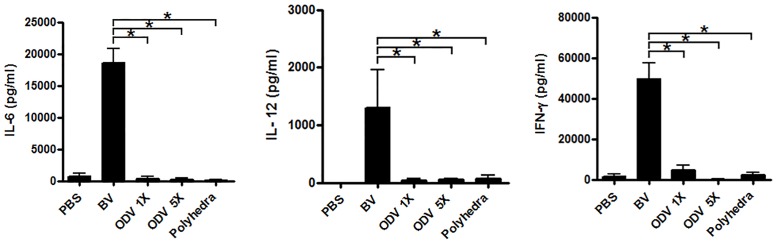
ODVs do not stimulate innate immune response in mice. C57BL/6 mice were i.v. injected with PBS, BVs, ODVs at two different concentrations or with polyhedra. Subsequently, sera were collected at 6 h. IL-6, IL-12 and IFN-γ levels were titrated by ELISA. The results are representative of two independent experiments. *, p<0.05.

Firstly, we evaluated the impact of ODVs on the murine innate immune system. BVs are known to promote the secretion of IFN-γ, IL-6 and IL-12 in mice. With this in mind we analized the profiles of these cytokines when induced by ODVs and polyhedra and compared the results to the profiles induced by BVs. We immunized C57BL/6J mice via i.v. with PBS, BVs, ODVs and ODV-containing polyhedra, and subsequently extracted serum samples 6 or 24 h after injection. BVs induced high levels of IL-6, IL-12 and IFN-γ but these cytokines were undetectable at 6 h post injection in sera from mice injected with ODVs or polyhedra (Fig 2A). IFN-γ was undetectable at 24 h in all of the groups (S1 Fig).

The innate response promoted by BV exposure leads to an antiviral state in the short term which protects mice or cell cultures against certain viral infections. To evaluate if the virions of both phenotypes could induce this state, we assessed the antiviral activity against VSV by titrating pools of the sera obtained at 6 h post exposure to this virus in IBRS-2 cells. While the pool of sera from BV-immunized mice exhibited an IC_50_ value of 2.21%, the pool from ODV- immunized mice were unable to protect the cells at 25%, the lower serum dilution assessed (Table 1).

**Table 1:** ODVs do not promote antiviral activity in mice.

Inoculum	IC_50_ (% v/v)^a^
**PBS**	> 25% serum^b^
**BV**	2.21% serum
**ODV 1X**	> 25% serum
**ODV 5X**	> 25% serum

C57BL/6 mice were i.v. injected with PBS, BVs, ODVs in two different concentrations and sera were collected at 6 h. Antiviral activity in serum was assessed. IBRS-2 cells were incubated with serial dilutions of pools of sera for 18 h and infected with stomatitis vesicular virus. The results are representative of two independent experiments.

^a^Half maximal inhibitory concentrations of the infection (IC_50_) expressed as % (v/v).

^b^The minimal dilution assessed_._

**Fig 3:**
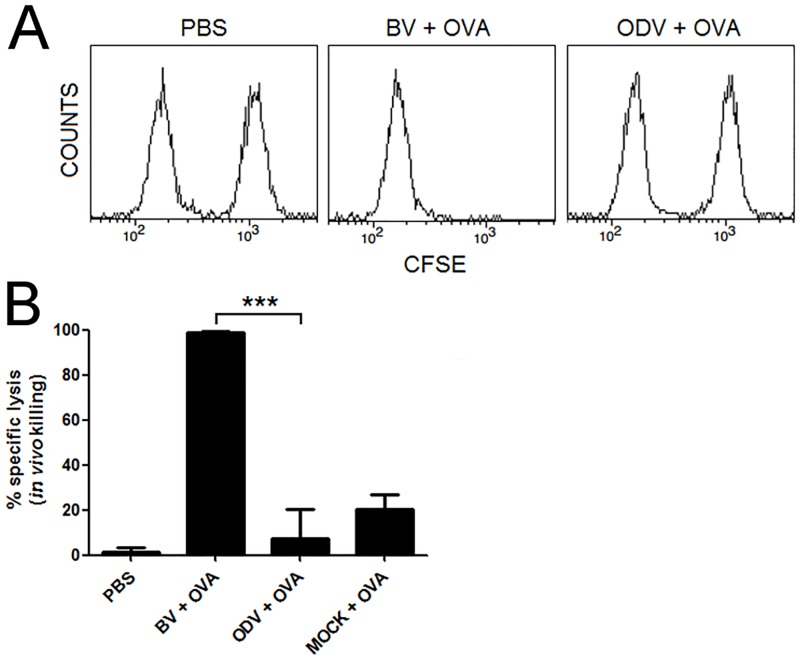
ODVs do not elicit cellular response against a coadministered antigen. C57BL/6 mice were immunized by a single i.v. injection of PBS or OVA (1 mg) combined with BVs, ODVs or mock purification of ODVs. Seven days later, immunized mice received an i.v. injection of a mixture (1:1) of OVA_256-264_peptide-loaded CFSE^high^ and unloaded CFSE^low^ splenocytes as target cells. (A) A representative histogram of remaining CFSE^high^ and CFSE^low^ cells in control, ODVs+OVA and BVs+OVA immunized mice 20 h after injection of target cells is shown. (B) Percentage of specific *in vivo* killing. ***, p<0.001. The results are expressed as mean +/− SEM (n = 4).

BVs can induce cytotoxic responses as well as the production of IFN-γ by CD8 and CD4 T cells specific to the co-administered antigen. With this in mind, we assessed putative ODV adjuvant properties. Firstly, we immunized C57BL/6J mice via i.v. with 1 mg of OVA alone or combined with ODVs, BVs or a mock purification of ODVs. Seven days later, we determined the CTL specific response to the OVA_257-264_ epitope by an *in vivo* killing assay with CFSE-labeled target cells. BVs elicited 98.6% of OVA-specific lysis, whereas equivalent quantities of ODVs exhibiting the same VP39 mass failed to induce any detectable CTL response (Fig 3). Additionally, we restimulated spleen cells from the immunized mice with OVA, and after 48 h analyzed the presence of IFN-γ in pools of their supernatants by ELISA. Splenocytes of mice immunized with OVA mixed with BVs displayed high levels of IFN-γ. By contrast, no IFN-γ was detected in pooled supernatants from the other groups (S2 Fig).

### ODVs are able to enter BMDCs and reach late and acidic endosomes

**Fig 4:**
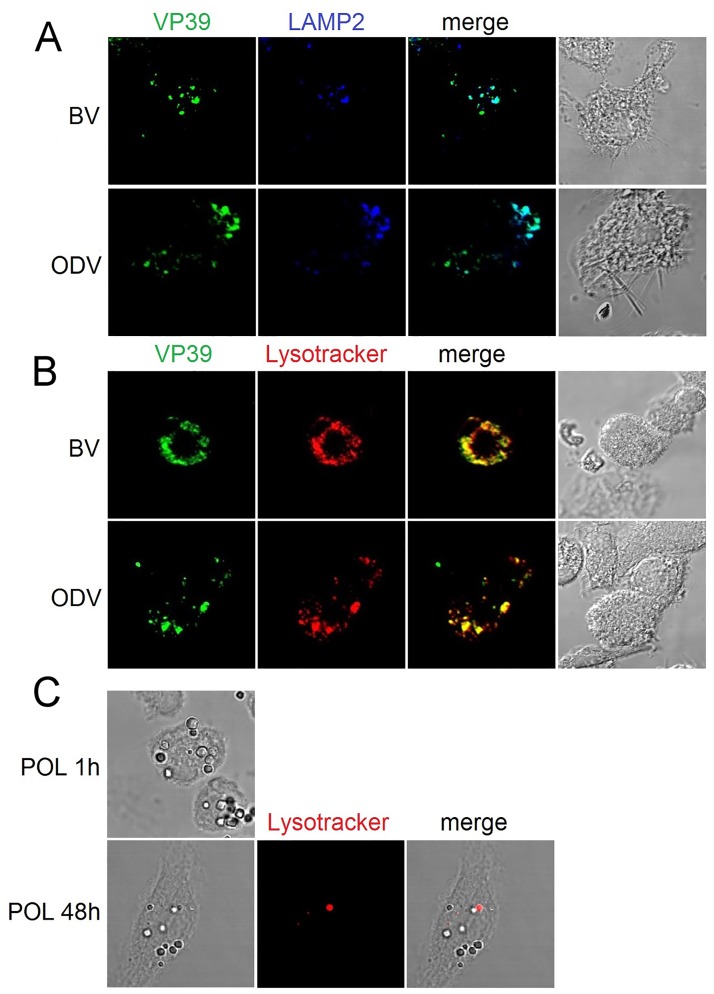
ODVs reach late and acidic endosomes in BMDCs. BVs or ODVs were incubated with immature BMDCs at 37°C (A) for 1 h or (B) for 1:15 h and then fixed, permeabilized and stained with specific antibodies. (C) Polyhedra were incubated with immature BMDCs at 37°C for 1 or 48 h, and treated in the same way. All the analyses were carried out by confocal microscopy. LysoTracker DND-90 is shown in red, VP39 in green and LAMP2 in blue.

DCs have a pivotal role in the immune response. Indeed, they are responsible for recognizing PAMPs by their receptors and for directing the profile of the adaptive immune response. Therefore, we compared the entrance and the traffic of ODVs and BVs in BMDCs by immunofluorescence staining and confocal microscopy analysis. Both BVs and ODVs entered the cells and 1 h post-infection they were inside LAMP2+ vesicles (Fig 4A). This result suggests that BVs and ODVs are able to reach late endosomes or lysosomes. Furthermore, BVs and ODVs colocalized with LysoTracker, a marker of acidic compartments (Fig 4B).

Additionally, we investigated the ability of DCs to phagocyte occlusion bodies by incubating BMDCs and polyhedra. According to the immunofluorescence microscopy assays, occlusion bodies were endocytosed by BMDCs 1 h after incubation; 48 h later they were still detectable inside the cells, although some of them were in LysoTracker+ compartments (Fig 4C).

### ODVs do not induce BMDCs maturation

**Fig 5:**
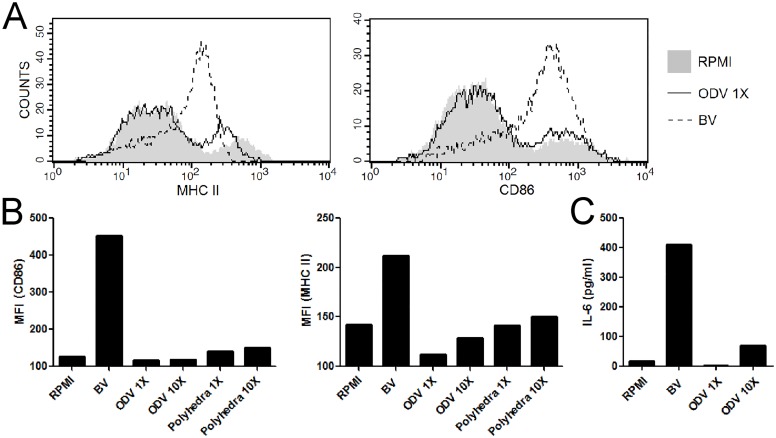
ODVs do not induce BMDCs maturation. BMDCs were incubated for 18 h with RPMI medium alone, BVs, ODVs or polyhedra. Cells were labeled with anti-CD11c, MHCII and CD86, and analyzed on a FACSCalibur. The results are representative of two independent experiments and are expressed (A) in histogram and (B) in bars graph as the geometric mean of the fluorescence intensity (MFI) for each indicated maturation marker in total CD11c+ cells (conventional DCs). (C) IL-6 levels were determined in BMDC supernatants by ELISA.

BVs activate BMDCs and in turn stimulate the expression of surface activation markers and the production of inflammatory cytokines. We investigated if the occluded phenotype is equally capable of inducing the same activation. Therefore, we incubated BMDCs with ODVs, polyhedra, BVs and mock for 18 h and subsequently evaluated the increment of surface expression of class II major histocompatibility complex molecules (MHC II) and the costimulatory molecule CD86, which are markers of activation. Thus, whereas BVs up-regulated BMDCs’ surface markers, ODVs or polyhedra did not induce these markers (Fig 5A and 5B). In fact, the cells infected with ODVs or polyhedra showed a similar profile to that of the mock treated cells.

To complete this characterization, we analyzed the inflammatory cytokine IL-6 by ELISA. Unlike BVs, ODVs failed to promote the secretion of this cytokine (Fig 5C).

### BVs but not ODVs share their fate in BMDCs with OVA

**Fig 6:**
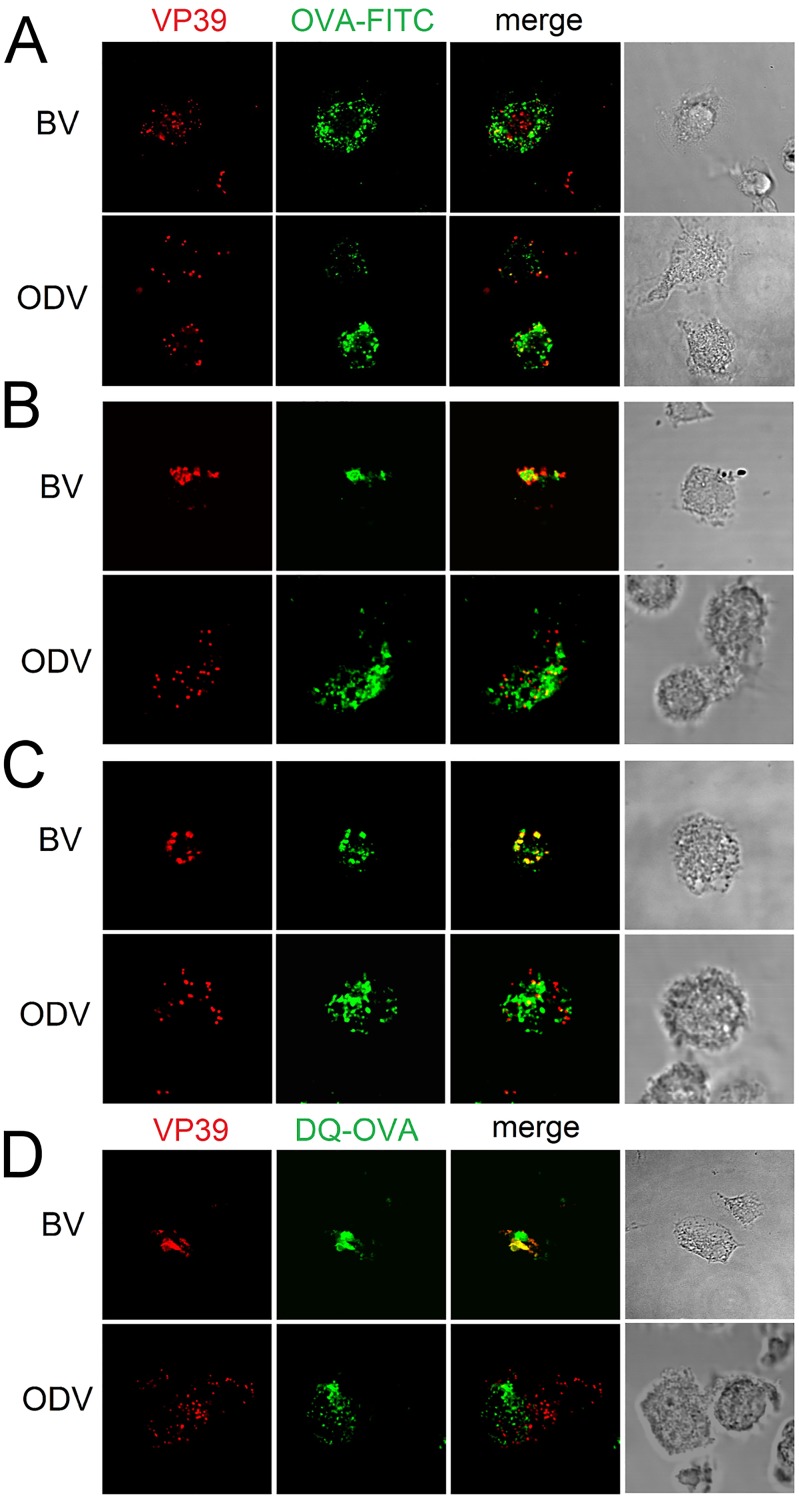
BVs but not ODVs share their fate in BMDCs with OVA. Immature BMDCs were incubated with either BVs or ODVs together with OVA-FITC at 37°C for (A) 10 min, (B) 40 min or (C) 75 min or (D) with DQ-OVA for 3 h. After the fixation and permeabilization they were marked with specific antibodies. VP39 is shown in red and OVA-FITC or DQ-OVA in green.

According to our observations in BMDCs, the model antigen ovalbumin and BVs are internalized in individual vesicles (Fig 6A and 6B) and then received by a common endosomal compartment (Fig 6C). With this in mind, we used this antigen for comparing the fate of both baculoviral phenotypes. For this purpose, we incubated BMDCs with OVA-FITC and BVs or ODVs, then revealed the nucleocapsids with anti-VP39 mAb and analyzed their colocalization by confocal microscopy. Only the budded virions colocalized markedly with OVA and not earlier than 75 min after the beginning of the incubation (Fig 6A, 6B and 6C). Then, we incubated BMDCs with each baculoviral phenotype and DQ-OVA for 3 h and subsequently marked the nucleocapsids in the same way. Unlike ODVs, BVs strongly colocalized with DQ-OVA. This finding indicated BVs localized inside degradative compartments (Fig 6D). Altogether, these results demonstrated that BVs share their destiny with OVA, whereas ODVs follow a different endosomal traffic under these experimental conditions.

## Discussion

Baculoviruses have interesting properties as viral vectors. For example, they are non-pathogenic to humans, allow large heterologous sequences in the genome, and present low cytotoxicity. In addition, BVs can transduce a broad range of mammalian cells. The effects on the immune system give them a particular potential as antiviral and anticancer agents and in the development of immunization strategies, although they have also been assessed in gene therapy [6, 16–19]. The use of recombinant BVs carrying chimerical proteins in the surface (baculovirus display) or the capsid (capsid display) as an immunization strategy was successfully used to induce humoral and cellular immune responses against several pathogens [16, 20–27].

ODVs are produced in large quantities in cell cultures and ODV production and purification would offer important advantages for the industry [28]. However, the interaction of occluded phenotype with vertebrates has been poorly studied. For many applications, ODVs may have similar effects to those of BVs. In this work, we focused on investigating the immune response elicited by ODVs respect to BVs.

To evaluate the immune properties of ODVs, we first studied their effect *in vivo*. BVs administrated intravenously are taken up by the liver and spleen, infect DCs and B lymphocytes and in turn induce high levels of inflammatory cytokines within 6 h post injection [29]. By contrast, ODVs injected by the same via were unable to promote inflammatory cytokines or antiviral activity at short or long terms. Furthermore, ODVs failed to exert any immunostimulatory response as adjuvant, unlike BVs, when co-administered with ovalbumin.

DCs show high endocytic ability and have a pivotal role both in innate and adaptive immune response. For this reason, we focused our study on the effect of polyhedra and ODVs in these cells. Polyhedra were efficiently captured and localized in acidic compartments of DCs, but were not degraded. This could be explained because polyhedra require an extremely alkaline medium for their dissolution. Indeed, in the mid-gut of lepidopteran larvae, where polyhedra naturally dissolve, the medium is highly alkaline. Interestingly, polyhedra of *Anticarsia gemmatalis nucleopolyhedrovirus*, but not of AcMNPV, were able to modulate the immune system in mice under certain conditions [30]. On the other hand, ODVs entered in DCs and colocalized with acidic and Lamp 2+ compartments, thus demonstrating that virions of the occluded phenotype reached late endosomes and lysosomes similarly to virions of the budded phenotype.

BVs activate DCs and induce them to produce inflammatory cytokines and type II IFNs via the endocytic TLR9/MyD88 dependent pathway. Virions of this phenotype also induce these cells to produce type I and II IFNs via STING cytosolic DNA sensing pathway. ODVs share the same DNA genome with BVs and reached late endocytic compartments. However, ODVs neither maturate BMDCs nor induce the production of inflammatory cytokines and seem to be incapable of stimulating the TLR9/MyD88 dependent pathway. Similar results were obtained with ODVs of *Antheraea pernyi nucleopolyhedrovirus* in a chicken macrophage cell line but its DNA succeeded in promoting IL-12 and IFN-γ once mixed with liposome [9]. This finding indicates the functionality of their PAMPs and a misslocation between the agonist and its receptor.

We further studied the traffic of both budded and occlusion-derived virions by using soluble OVA as a model of exogenous antigen to detect any distinctiveness in the transit of both phenotypes inside DCs. Only BVs colocalized with soluble OVA, thus suggesting differences in their transit that could explain the lack of stimulation by ODVs. Similarly, we detected BVs only in degradative vesicles of DCs as evidenced by their co-localization with DQ-OVA. By contrast, no colocalization of ODVs with DQ-OVA was detected in degradative vesicles, which is a condition required for proper activation of the TLR 9 pathway [31, 32].

Despite BVs and ODVs share the DNA genome, they differ in several structural components, as tegument, nucleocapsid and surface proteins [33]. Surface proteins are the responsible for primary interactions with cellular ligands initiating a cascade of events that modulates the destiny of the virion [34]. Furthermore, both phenotypes differ in the origin and nature of their envelope. So, these differences could explain the lack of impact on the immune response. Abe and colleagues demonstrated that GP64, the major glycoprotein of BV, is an essential element for the activation of the immune response induced by AcMNPV. Indeed, BVs lacking this glycoprotein were incapable of stimulating the secretion of TNF-alpha in the murine macrophage cell line RAW264.7 [6]. ODVs have a complex envelope composition and can enter vertebrate cell lines [10,11]. We suggest that the lack of GP64 or the presence of *per os* infection factors in ODVs is responsible for the destiny towards a different endosome, where ODVs cannot activate the TLR 9 pathway. These factors would also impair ODV escape to the cytoplasm, thus compromising the activation of the STING pathway in DCs. However, we cannot discard that differences in structural components other than surface proteins can influence the final composition of endosomes.

Altogether, occlusion bodies and purified ODVs of AcMNPV enter and reach acidic compartments in DCs similarly to BVs. However, virions of this phenotype are unable to impact on the murine immune system under the studied conditions. Due to the low immunogenicity of ODVs, virions of this phenotype could be improved by genetic engineering to become useful tools for gene delivery or, due to their particulate structure, as vaccine vectors with appropriate adjuvants.

## Supporting Information

S1 Fig ODVs do not stimulate innate immune response in mice. C57BL/6 mice were i.v. injected with PBS, BVs, ODVs in two different concentrations or polyhedra. Subsequently, sera were collected at 6 and 24 h. IL-6, IL-12 and IFN-γ levels were titrated by ELISA. The results are representative of two independent experiments. *, p<0.05.
(TIFF)

S2 Fig ODVs do not elicit cellular response against coadministered antigen. C57BL/6 mice were immunized by a single i.v. injection of PBS or OVA (1 mg) combined with BVs, ODVs or mock purification of ODVs. Seven days later, splenocytes from immunized mice were cultured and restimulated with OVA. Subsequently, supernatants were collected at 48 h and IFN-γ levels were titrated by ELISA. The results are representative of two independent experiments.
(TIFF)

## References

[1] Rohrmann GF. Baculovirus Molecular Biology. 3rd ed Bethesda (MD) 2013. 24479205

[2] Braunagel SC, Russell WK, Rosas-Acosta G, Russell DH, Summers MD. Determination of the protein composition of the occlusion-derived virus of Autographa californica nucleopolyhedrovirus. Proceedings of the National Academy of Sciences of the United States of America. 2003;100(17):9797–802. Epub 2003/08/09. 10.1073/pnas.1733972100 12904572 PMC187845

[3] Hu YC. Baculoviral vectors for gene delivery: a review. Current gene therapy. 2008;8(1):54–65. 18336250 10.2174/156652308783688509

[4] Oker-Blom C, Airenne KJ, Grabherr R. Baculovirus display strategies: Emerging tools for eukaryotic libraries and gene delivery. Brief Funct Genomic Proteomic. 2003;2(3):244–53. 15239927 10.1093/bfgp/2.3.244

[5] Tani H, Limn CK, Yap CC, Onishi M, Nozaki M, Nishimune Y, et al In vitro and in vivo gene delivery by recombinant baculoviruses. Journal of virology. 2003;77(18):9799–808. 10.1128/JVI.77.18.9799-9808.2003 12941888 PMC224587

[6] Abe T, Hemmi H, Miyamoto H, Moriishi K, Tamura S, Takaku H, et al Involvement of the Toll-like receptor 9 signaling pathway in the induction of innate immunity by baculovirus. Journal of virology. 2005;79(5):2847–58. 10.1128/JVI.79.5.2847-2858.2005 15709004 PMC548444

[7] Hervas-Stubbs S, Riezu-Boj JI, Mancheno U, Rueda P, Lopez L, Alignani D, et al Conventional but not plasmacytoid dendritic cells foster the systemic virus-induced type I IFN response needed for efficient CD8 T cell priming. J Immunol. 2014;193(3):1151–61. Epub 2014/06/29. 10.4049/jimmunol.1301440 24973449 PMC4105236

[8] Mogensen TH. Pathogen recognition and inflammatory signaling in innate immune defenses. Clinical microbiology reviews. 2009;22(2):240–73, Table of Contents. Epub 2009/04/16. 10.1128/CMR.00046-08 19366914 PMC2668232

[9] Niu M, Han Y, Li W. Baculovirus up-regulates antiviral systems and induces protection against infectious bronchitis virus challenge in neonatal chicken. International immunopharmacology. 2008;8(12):1609–15. 10.1016/j.intimp.2008.07.004 18707025

[10] Makela AR, Tuusa JE, Volkman LE, Oker-Blom C. Occlusion-derived baculovirus: interaction with human cells and evaluation of the envelope protein P74 as a surface display platform. Journal of biotechnology. 2008;135(2):145–56. Epub 2008/05/13. 10.1016/j.jbiotec.2008.03.014 18471919

[11] Volkman LE. Occluded and Budded Autographa californica Nuclear Polyhedrosis Virus: Immunological Relatedness of Structural Proteins. Journal of virology. 1983;46(1):221–9. 16789239 10.1128/jvi.46.1.221-229.1983 PMC255111

[12] Whitt MA, Manning JS. A phosphorylated 34-kDa protein and a subpopulation of polyhedrin are thiol linked to the carbohydrate layer surrounding a baculovirus occlusion body. Virology. 1988;163(1):33–42. Epub 1988/03/01. 3279702 10.1016/0042-6822(88)90231-0

[13] Faulkner P, Kuzio J, Williams GV, Wilson JA. Analysis of p74, a PDV envelope protein of Autographa californica nucleopolyhedrovirus required for occlusion body infectivity in vivo. The Journal of general virology. 1997;78 (Pt 12):3091–100. Epub 1997/12/24. 9400957 10.1099/0022-1317-78-12-3091

[14] Inaba K, Inaba M, Romani N, Aya H, Deguchi M, Ikehara S, et al Generation of large numbers of dendritic cells from mouse bone marrow cultures supplemented with granulocyte/macrophage colony-stimulating factor. The Journal of experimental medicine. 1992;176(6):1693–702. 1460426 10.1084/jem.176.6.1693 PMC2119469

[15] Moron G, Rueda P, Casal I, Leclerc C. CD8alpha- CD11b+ dendritic cells present exogenous virus-like particles to CD8+ T cells and subsequently express CD8alpha and CD205 molecules. The Journal of experimental medicine. 2002;195(10):1233–45. 10.1084/jem.20011930 12021304 PMC2193750

[16] Abe T, Takahashi H, Hamazaki H, Miyano-Kurosaki N, Matsuura Y, Takaku H. Baculovirus induces an innate immune response and confers protection from lethal influenza virus infection in mice. J Immunol. 2003;171(3):1133–9. 12874198 10.4049/jimmunol.171.3.1133

[17] Gronowski AM, Hilbert DM, Sheehan KC, Garotta G, Schreiber RD. Baculovirus stimulates antiviral effects in mammalian cells. Journal of virology. 1999;73(12):9944–51. 10559307 10.1128/jvi.73.12.9944-9951.1999 PMC113044

[18] Hervas-Stubbs S, Rueda P, Lopez L, Leclerc C. Insect baculoviruses strongly potentiate adaptive immune responses by inducing type I IFN. J Immunol. 2007;178(4):2361–9. 17277142 10.4049/jimmunol.178.4.2361

[19] Chen CY, Lin CY, Chen GY, Hu YC. Baculovirus as a gene delivery vector: recent understandings of molecular alterations in transduced cells and latest applications. Biotechnology advances. 2011;29(6):618–31. Epub 2011/05/10. 10.1016/j.biotechadv.2011.04.004 21550393 PMC7126054

[20] Molinari P, Crespo MI, Gravisaco MJ, Taboga O, Moron G. Baculovirus capsid display potentiates OVA cytotoxic and innate immune responses. PloS one. 2011;6(8):e24108 Epub 2011/09/16. 10.1371/journal.pone.0024108 21918683 PMC3168877

[21] Peralta A, Molinari P, Conte-Grand D, Calamante G, Taboga O. A chimeric baculovirus displaying bovine herpesvirus-1 (BHV-1) glycoprotein D on its surface and their immunological properties. Appl Microbiol Biotechnol. 2007;75(2):407–14. 10.1007/s00253-006-0825-4 17285288

[22] Strauss R, Huser A, Ni S, Tuve S, Kiviat N, Sow PS, et al Baculovirus-based Vaccination Vectors Allow for Efficient Induction of Immune Responses Against Plasmodium falciparum Circumsporozoite Protein. Molecular therapy: the journal of the American Society of Gene Therapy. 2007;15(1):193–202. 17164791 10.1038/sj.mt.6300008

[23] Tami C, Farber M, Palma EL, Taboga O. Presentation of antigenic sites from foot-and-mouth disease virus on the surface of baculovirus and in the membrane of infected cells. Archives of virology. 2000;145(9):1815–28. 11043943 10.1007/s007050070058

[24] Tami C, Peralta A, Barbieri R, Berinstein A, Carrillo E, Taboga O. Immunological properties of FMDV-gP64 fusion proteins expressed on SF9 cell and baculovirus surfaces. Vaccine. 2004;23(6):840–5. 10.1016/j.vaccine.2004.03.070 15542209

[25] Yang DG, Chung YC, Lai YK, Lai CW, Liu HJ, Hu YC. Corrigendum to "avian influenza virus hemagglutinin display on baculovirus envelope: cytoplasmic domain affects virus properties and vaccine potential". Molecular therapy: the journal of the American Society of Gene Therapy. 2007;15(9):1736. 10.1038/mt.sj.6300131 17375072

[26] Yang DG, Chung YC, Lai YK, Lai CW, Liu HJ, Hu YC. Avian influenza virus hemagglutinin display on baculovirus envelope: cytoplasmic domain affects virus properties and vaccine potential. Molecular therapy: the journal of the American Society of Gene Therapy. 2007;15(5):989–96. 17375072 10.1038/mt.sj.6300131

[27] Yoshida S, Kondoh D, Arai E, Matsuoka H, Seki C, Tanaka T, et al Baculovirus virions displaying Plasmodium berghei circumsporozoite protein protect mice against malaria sporozoite infection. Virology. 2003;316(1):161–70. 14599800 10.1016/j.virol.2003.08.003

[28] Claus Juan D. G VV, Micheloud Gabriela A. and Visnovsky Gabriel. Production of Insecticidal Baculoviruses in Insect Cell Cultures: Potential and Limitations, Insecticides—Basic and Other Applications. Soloneski DS, editor2012.

[29] Kitajima M, Abe T, Miyano-Kurosaki N, Taniguchi M, Nakayama T, Takaku H. Induction of Natural Killer Cell-dependent Antitumor Immunity by the Autographa californica Multiple Nuclear Polyhedrosis Virus. Molecular therapy: the journal of the American Society of Gene Therapy. 2008;16(2):261–8. 18059370 10.1038/sj.mt.6300364

[30] Bocca AL, Barros MC, Martins GK, de Araujo AC, Souza MJ, Ribeiro AM, et al Immunological effects of Anticarsia gemmatalis multiple nucleopolyhedrovirus (AgMNPV) by stimulation of mice in vivo and in vitro. Virus research. 2013;176(1–2):119–27. Epub 2013/06/12. 10.1016/j.virusres.2013.05.015 23747526

[31] Hacker H, Mischak H, Miethke T, Liptay S, Schmid R, Sparwasser T, et al CpG-DNA-specific activation of antigen-presenting cells requires stress kinase activity and is preceded by non-specific endocytosis and endosomal maturation. The EMBO journal. 1998;17(21):6230–40. Epub 1998/11/03. 10.1093/emboj/17.21.6230 9799232 PMC1170949

[32] Park B, Brinkmann MM, Spooner E, Lee CC, Kim YM, Ploegh HL. Proteolytic cleavage in an endolysosomal compartment is required for activation of Toll-like receptor 9. Nature immunology. 2008;9(12):1407–14. Epub 2008/10/22. 10.1038/ni.1669 18931679 PMC2735466

[33] Hou D, Zhang L, Deng F, Fang W, Wang R, Liu X, et al Comparative proteomics reveal fundamental structural and functional differences between the two progeny phenotypes of a baculovirus. Journal of virology. 2013;87(2):829–39. Epub 2012/11/02. 10.1128/JVI.02329-12 23115289 PMC3554090

[34] Vazquez-Calvo A, Saiz JC, McCullough KC, Sobrino F, Martin-Acebes MA. Acid-dependent viral entry. Virus research. 2012;167(2):125–37. Epub 2012/06/12. 10.1016/j.virusres.2012.05.024 22683298

