# Supplementary figures and images for: Two Distinctive Phenotypes of AcMNPV Display Different Immune Abilities and Intracellular Destiny
Source: PLoS One. 2016 Dec 29;11(12):e0168939. doi: 10.1371/journal.pone.0168939 (PMC5199047; doi:10.1371/journal.pone.0168939)

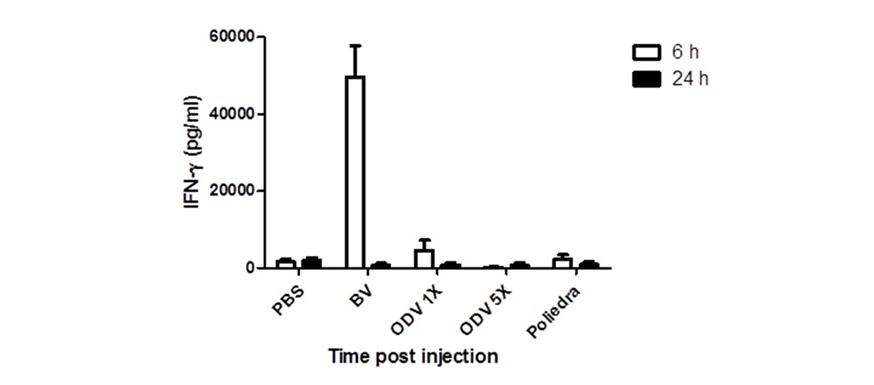

Supplement: S1 Fig — C57BL/6 mice were i.v. injected with PBS, BVs, ODVs in two different concentrations or polyhedra. Subsequently, sera were collected at 6 and 24 h. IL-6, IL-12 and IFN-γ levels were titrated by ELISA. The results are representative of two independent experiments. *, p<0.05. (TIFF) [file pone.0168939.s001.tiff]

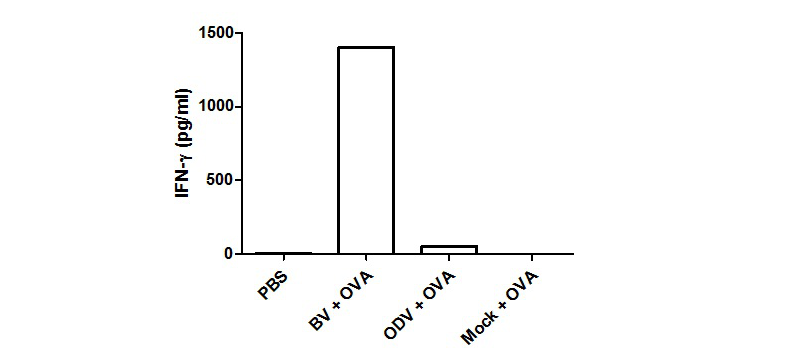

Supplement: S2 Fig — C57BL/6 mice were immunized by a single i.v. injection of PBS or OVA (1 mg) combined with BVs, ODVs or mock purification of ODVs. Seven days later, splenocytes from immunized mice were cultured and restimulated with OVA. Subsequently, supernatants were collected at 48 h and IFN-γ levels were titrated by ELISA. The results are representative of two independent experiments. (TIFF) [file pone.0168939.s002.tiff]
